# Impact of traditional Chinese Baduanjin exercise on menstrual health among international female students studying in China: a randomized controlled trial

**DOI:** 10.3389/fpubh.2024.1259634

**Published:** 2024-02-07

**Authors:** Asem Alkhatib, Hoda Alshikh Ahmad, Ci Zhang, Wenwen Peng, Xianhong Li

**Affiliations:** ^1^Xiangya School of Nursing, Central South University, Changsha, Hunan, China; ^2^Xiangya School of Public Health, Central South University, Changsha, Hunan, China

**Keywords:** acculturation, Baduanjin, international students, menstrual health, symptoms

## Abstract

**Background:**

Menstrual symptoms were the common complaints of international female students during the acculturation period, and the symptoms negatively affected the daily life and school performance of those women. The present study aimed to evaluate the effectiveness of the traditional Chinese Baduanjin exercise on reducing menstrual symptoms for international female students studying in China during the acculturation period.

**Methods:**

We conducted a randomized controlled trial among 62 international female students who suffered menstrual symptoms during the first 6 months after arriving in China. The study was carried out at three campuses of the two largest universities in the capital of a province in south-central China from March to October 2021. After screening, participants were randomly assigned to a control or intervention group. The intervention group engaged in 24 weeks of Baduanjin exercise for at least 30 min per day. The menstrual symptoms, sociocultural adaptation, perceived stress, and sleep quality were evaluated at baseline, the 12th week, and the 24th week. The chi-square test or Fisher's exact test, paired sample *t*-test, and multivariate analysis of variance (MANOVA)were adopted to analyze the data using SPSS 25.0.

**Results:**

Totally, 62 participants were enrolled in this study from 30 countries. Their mean age was 27.87 ± 5.58 years. None of the participants reported an adverse event. The results of the MANOVA test showed that the traditional Chinese Baduanjin exercise reduced the total score of MSQ among international female students (intervention: 47.83 ± 8.71 vs. control: 65.12 ± 16.86; *F* = 19.49, *P* < 0.01). In addition, the results of the MANOVA indicated statistically significant decreases in scores for the two subscales of MSQ: premenstrual symptoms (intervention: 17.07 ± 3.33 vs. control: 28.42 ± 7.56; *F* = 44.70, *P* < 0.01)and menstrual pain (16.03 ± 4.53 vs. 19.58 ± 5.14; *F* = 6.50, *P* < 0.05). Moreover, the results showed that traditional Chinese Baduanjin exercise reduced the scores of the sociocultural adaptation level, the perceived stress level, and sleep quality and improved the blood progesterone hormone and blood estrogen hormone.

**Conclusion:**

Regular Baduanjin exercise is a safe, acceptable, and effective form of exercise to promote international female students' menstrual health, reduce stress levels, and improve sleep quality.

**Trial registration:**

www.chictr.org.cn, Chinese Clinical Trial Registry: ChiCTR2300072376.

## 1 Introduction

Globalization has changed all aspects of life in recent decades. Education in particular has become more international, with 4.78 million students studying abroad each year (1). In recent years, China has become one of the most popular destinations, and the number of international students studying and living in China has significantly increased from 223,499 in 2008 to 492,185 in 2018 (2). Studying in a foreign country involves a process of acculturation, which can affect the social and psychological wellbeing of international students (3). Although many studies have examined the consequences of acculturation for international students, such as culture shock, psychological distress, anxiety, and withdrawal behaviors in seeking help (4, 5), only a few have focused on the international students' menstrual health disturbances caused by the acculturation (6).

Menstruation is a normal physiological phenomenon that can be affected by the environment, weather, nutrition, lifestyle (such as sociocultural adaptation), blood hormone levels (such as progesterone, estrogen, and prolactin), and psychological pressures (7, 8). Previous studies found that menstrual irregularities occurred among the women after they relocated to a new country (9–11). Our team's previous study also reported that 18.49% of international female students experienced a change in their menstrual symptoms after arriving in China (6). Generally, mild menstrual symptoms do not affect women's life or work (12). Still, troublesome experiences of menstruation, such as severe menstrual pain associated with poor sleep quality (13), could negatively affect a woman's daily life and work (14). Our previous qualitative interviews indicated that international female students reported missing classes or examinations due to the severe menstrual symptoms caused by acculturation (15). Therefore, the menstrual health of international female students has become an important concern that needs to be addressed by school educators and healthcare providers.

Current evidence clearly shows that exercise or physical activity is effective in relieving menstrual symptoms and promoting menstrual health in women (16). A recent meta-analysis based on a systematic review of randomized controlled trials indicated that general aerobic exercises could reduce depression and anxiety symptoms, improve self-esteem, and increase menstruation-related hormone levels, such as progesterone, estrogen, and prolactin (17). Moreover, Mohebbi Dehnavi's study also indicated that general aerobic exercise could significantly decrease the physical symptoms of headache, nausea, constipation, diarrhea, and swelling during women's menstrual period (18). Moreover, Jang's study in China showed that Qigong therapy (a traditional Chinese aerobic exercise)could reduce premenstrual symptoms of pain, negative feelings, and water retention (19). Çitil's study using Pilates exercises found that it could relieve depressive symptoms, anxiety, irritability, fatigue, pain, changes in appetite, changes in sleep, and swelling during the menstrual period (20), and Günebakan's study confirmed Yoga exercises' effectiveness in reducing somatic discomfort and menstrual pain and improving women's coping strategies (21).

As a traditional aerobic exercise for body and mind in China, Baduanjin is one of the most common forms of traditional Chinese exercise and has been practiced for over 1,000 years. It has repetitive movements of low intensity and meditation (22), and it integrates using breathing techniques in a harmonious way (23). Many studies have reported that Baduanjin exercise has a positive effect on improving physical functions, for example, flexibility, physical fitness, muscle strength, and cardiorespiratory endurance (24), and it could also improve sleep quality (25), mood status (26), quality of life (27), and general wellbeing (28). However, only two studies in the literature examined the effectiveness of Baduanjin in reducing menstrual symptoms. One was done among Chinese female college students and showed effectiveness in relieving the symptoms of primary dysmenorrhea (29); another study was conducted among Chinese women in Macau, and it also indicated significant improvements in reducing the general symptoms of premenstrual syndrome, especially the physical symptoms (30). Currently, there is no study examining this traditional Chinese exercise's impact on improving menstrual health, especially for international female students studying in China who are undergoing the acculturation period and hold different cultural beliefs. Therefore, our study aimed to examine the acceptability and impact of Baduanjin exercise on the menstrual health of international female students who were studying in China and enduring changed menstrual symptoms.

## 2 Materials and methods

### 2.1 Study design

The study was a two-arm randomized controlled trial and consisted of an experimental group (EG)and a control group (CG). This study was conducted from March to October 2021 and reported following the Consolidated Standards of Reporting Trials (CONSORT 2010 Checklist).

### 2.2 Participants

The study recruited international female students from three campuses of the two largest universities in Changsha, the capital city of Hunan Province, Mainland China. International female students who met the following criteria were recruited into the study: (a)aged 18 years and above; (b)had primary and secondary education in a foreign country or region outside of China; (c)were pursuing a bachelor's, master's, or doctoral degree in China; and (d)self-reported experiencing menstrual symptoms change after arriving in China. We defined the menstrual symptoms change as “changes in severity or frequency of symptoms such as cramps, irritability, pain, or fatigue that were not experienced before living in China, and the changes lasted at least two consecutive menstrual cycles before joining the study”; (e)understood Mandarin or English; and (f)would live in Hunan province for the following 6 months. Those who were pregnant, having a physical illness (diagnosed with gynecological disorders such as primary amenorrhea, polycystic ovary syndrome, and any pathological situations that could cause menstrual disturbances), taking any medication that could affect their menstrual symptoms, or having an injury that prevents them from exercising properly would be excluded.

### 2.3 Procedures

#### 2.3.1 Screening

Initially, official permission was obtained from the two universities, which allowed the study flier to be posted on their advertising boards. A flier was also sent electronically through WeChat (an online instant chat platform)groups established by each university for their international students. Those interested in participating in this study contacted the researcher's assistant, who examined their eligibility. The eligible participants made an appointment to meet with the research assistant to fully understand the study procedure, benefits, and potential risks and sign the informed consent; then they completed the baseline questionnaires and had blood tests in the designated hospital. After that, they were randomly assigned to either the experimental group (EG)or the control group (CG).

#### 2.3.2 Intervention procedure

Participants in the CG received an information sheet to elaborate on the influencing factors for menstrual symptoms changes and the potential useful exercises (e.g., aerobics, yoga, and Pilates)to relieve the symptoms, not including Baduanjin practice. Relevant web links to these exercises were sent to them for their practice based on their willingness.

Participants in the EG group had two phases of Baduanjin practice. The first phase was practicing Baduanjin in a group (~10–12 people)with a guided mentor once a day for 12 consecutive weeks. There were three groups located on the three campuses. Every day, they needed to exercise Baduanjin in a group for ~30 min. One of the guided mentors had more than 2 years of experience in practicing Baduanjin, and the other two mentors were graduate students who received Baduanjin training for 1 week and practiced for 2 months. The researcher assigned one participant in each group as a leader who was responsible for the participants' maintenance in practice by sending reminders every day through the WeChat group. Debriefings were also conducted in each group every week, led by the first author of this study. They could share their practicing experiences, feelings, thoughts, and some perceived body or mood changes, or any other confusion regarding practicing Baduanjin. They were also encouraged to share and discuss their thoughts or feelings in the WeChat group.

The second intervention phase was the exercise by the participants themselves without supervision for another 12 weeks. The participants in each group were asked to continue practicing with the same frequency as the supervised phase at an agreed-upon time and place. The research assistant sent reminders three times a week in the WeChat group to remind them of their practice and facilitate their debriefing and discussion in the WeChat group.

The whole procedure of the Baduanjin exercise was conducted according to the “Health Qigong Baduanjin Standard” published by the General Administration of Sports of China in 2003 (31). The whole set of Baduanjin exercises consists of 10 postures (including the preparation and ending postures). The eight postures (excluding the preparation and ending postures)are “holding the hands high with palms up to regulate the internal organs,” “posing as an archer shooting both left- and right-handed,” “holding one arm aloft to regulate the functions of the spleen and stomach,” “looking backwards to prevent sickness and strain,” “swinging the head lowering the body to relieve stress,” “moving the hands down the back and legs and touching the feet to strengthen the kidneys,” “thrusting the fists and making the eyes glare to enhance strength,” and “raising and lowering the heels to cure diseases.” This exercise set aims not only to reinforce musculoskeletal fitness and blood circulation along with breathing training but also to adjust emotions to achieve the fitness of the body and mind. The general postures were clearly explained and shown through many formal videos, such as the Chinese Health Qigong Association “YouTube channel” at https://www.youtube.com/watch?v=poxClwHOLgs&ab_channel=ChineseHealthQigongAssociation.

### 2.4 Data collection

Data were collected online through a specialized Chinese questionnaire platform (http://www.sojump.com), which could ensure the data's confidentiality and the participants' privacy. Links to the surveys were sent to the participants via WeChat. The questionnaire was collected at three time points of the baseline (T0), which was set before the start of the intervention, and the second time point (T1), which was set at the end of the supervised intervention phase (the 12th week), and the third time point (T2), which was set at the end of the program (the 24th week), and blood hormones were collected for the participants at the baseline and at the end of the program (the 24th week)only. Participants were taken to a designated hospital for blood tests, and only the required samples for these hormone tests were provided based on laboratory staff recommendations, while the rest of the samples were destroyed directly after completion of the tests. The research assistant explained the whole process of blood tests and the fate of the remaining blood samples to each participant in detail, then recorded the testing results, and sent them to each of them individually.

### 2.5 Outcomes and measures

The demographic characteristics assessed included age, marital status, home country, study information such as major, education program, enrollment year, source of funding for education, whether they have been to China before, and whether they have medical insurance.

The primary outcome was the menstrual symptoms, and it was assessed using the menstrual symptoms questionnaire (MSQ)(32). The MSQ was a 24-item self-reported scale originally developed to assess dysmenorrhea symptoms, and it has been widely used around the world. A 5-point Likert scale ranging from 1 (never)to 5 (always)was used as responses, with a higher overall score, indicating more severe menstrual symptoms. The reliability of MSQ designated by Cronbach's alpha was 0.93 (33), and in another study, Cronbach's alpha was 0.91 (34). According to Wildman and White's research (35), the MSQ scale was divided into three dimensions: premenstrual symptoms, psychophysiological discomfort, and menstrual pain, which are presented in Supplementary Table S1.

The secondary outcomes of the study were the sociocultural adaptation, stress, sleep quality, and blood hormone level, including progesterone and estrogen, and the outcomes were measured as follows:

1. Sociocultural adaptation was measured using the Sociocultural Adaptation Scale (SCAS), developed by Ward and Kennedy (36). The scale included 21 items, and each item was rated using a 5-point Likert scale ranging from 1 (no difficulty)to 5 (extreme difficulty). The average total score was used to evaluate the level of sociocultural adaptation, with a higher score indicating greater difficulty. Cronbach's alpha of the SCAS ranged from 0.75 to 0.91, as reported by a number of studies (36, 37).
2. Stress level was assessed using the Perceived Stress Scale (PSS)(38), which consisted of 10 items. Each item was rated on a 5-point Likert scale (0 = never, 4 = very often), with a higher score reflecting a greater perceived stress level. The PSS had an internal consistency designated by Cronbach's alpha of 0.78 (39).
3. Sleep quality was evaluated using the Pittsburgh Sleep Quality Index (PSQI)(40). The self-reported scale contained 19 individual items, creating seven components. Each item was rated from 0 (no difficulty)to 3 (severe difficulty). The global PSQI score was calculated by adding scores from the seven components, with a higher value indicating poorer sleep quality. The coefficient of Cronbach's alpha of PSQI was 0.83 (40).
4. Blood hormone tests (progesterone and estrogen)were performed using a chemiluminescent immunoassay method in the hospital.

### 2.6 Randomization and blinding

After screening, those eligible participants were invited to complete the baseline assessment, including the baseline questionnaire and blood hormones test, in the designated hospital. Then, a team member randomly divided the participants into two groups using a random number table generated using SPSS 25.0 (SPSS Inc., Chicago, IL, USA). The campuses were used to stratify the randomization. An identification code was assigned to each participant in the dataset, so the statistician was blinded to the groups.

### 2.7 Statistical analysis

Frequencies and percentages were used to describe the categorical variables, and means and standard deviations were used to describe the distributions of continuous variables. Categorical data were analyzed using the chi-square test (sample size no <40 and the expected number in each cell no <5)or Fisher's exact test (sample size <40 or the expected number in any one cell <5)(41). An independent sample *t*-test was conducted to compare the difference between the two study groups for the factors studied at baseline only among all the study variables. A paired sample *t*-test was performed to compare the difference between the two study groups for the factors examined at only two time points, such as blood hormones. A multivariate analysis of variance (MANOVA)was performed between the two study groups for all the primary and secondary outcomes among the three time points of this study, and the effects evaluated included the main effects of time and groups (EG and CG)as well as the interaction effect(s)of time by the groups. A *P*-value < 0.05 was considered statistically significant. All statistical analyses were performed using SPSS 25.0 (SPSS Inc., Chicago, IL, USA).

### 2.8 Sample size

Based on the previous study of the effect of Baduanjin exercises on menstrual symptoms (30), a sample size of 50 women would provide 80% power with an alpha of 0.05 to detect a medium to large statistically significant difference of 0.529. We calculated Cohen's *d* effect size, and threshold values were set as small (≥0.2 and <0.5), moderate (≥0.5 and <0.8), and large (≥0.8)(42). The mean difference between the menstrual symptoms was calculated using the data of Zhang's study as a reference (e.g., Cohen's *d* = mean of baseline—mean of last evaluation/baseline standard deviation), and based on the data of Zhang's study, the effect size was 0.529. Assuming a maximum loss to follow-up rate of 20%, the total sample size for this study is about 60 participants.

### 2.9 Ethical consideration

This study was approved by the Institutional Review Board of Behavioral and Nursing Research at the Xiangya School of Nursing of Central South University (Approval No. E2020193). Permission to conduct the study at the targeted universities was obtained from the School of International Education at each site. The study's purpose, duration, and procedures were fully explained to the participants, and written informed consent was obtained from all participants. Each participant participated in the study voluntarily and could withdraw without any consequences. The information obtained from the participants was confidential and was only being used for this study. After collecting the last-time-point data, we provided the video of the exercise to the control group participants. This study was carried out in accordance with relevant ethical guidelines and regulations of the Declaration of Helsinki.

### 2.10 Trial registration

This study was registered with the Chinese Clinical Trial Registry (www.chictr.org.cn, ID: ChiCTR2300072376).

## 3 Results

### 3.1 Demographic characteristics

**Figure 1:**
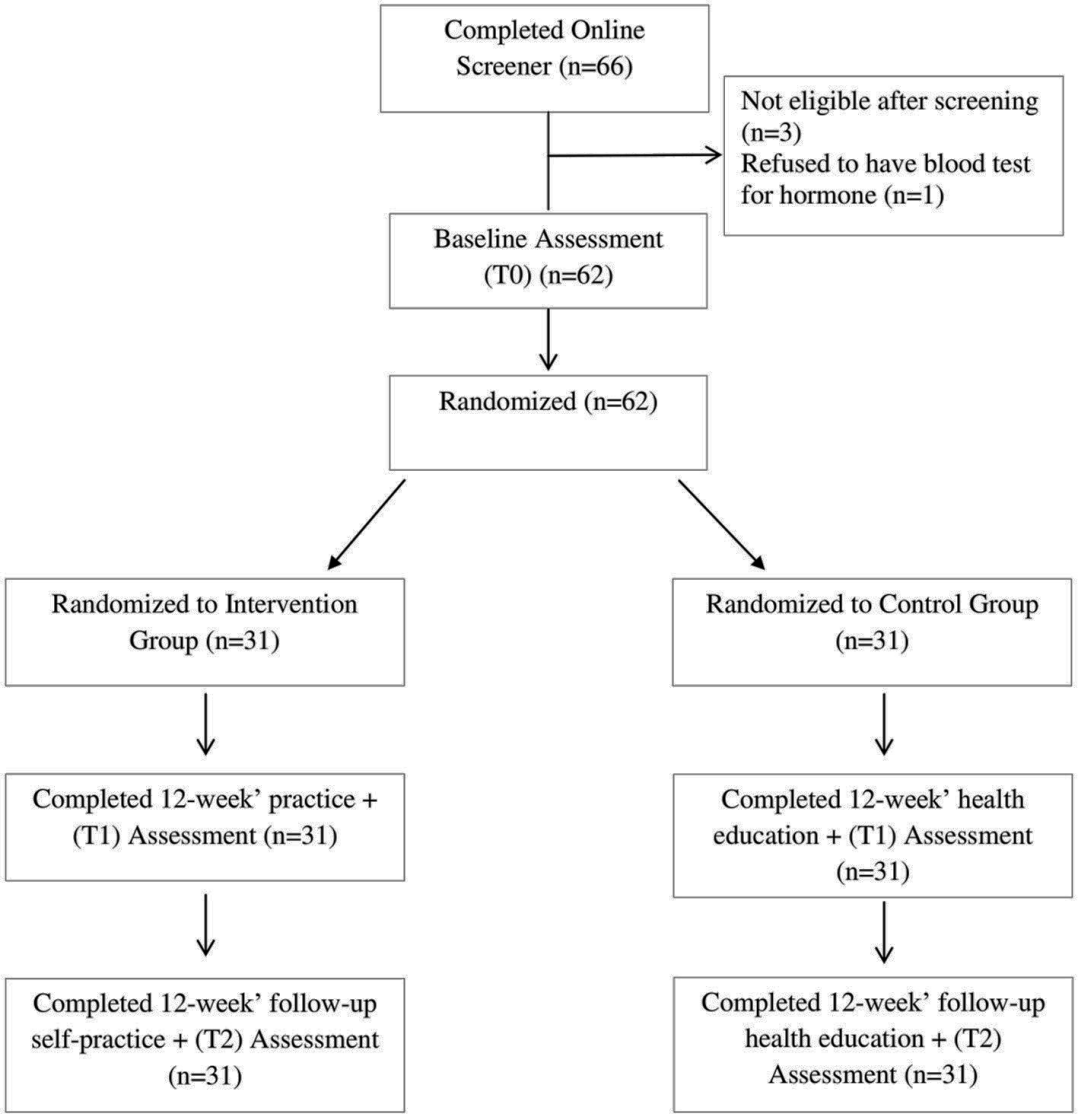
The study flowchart.

In total, 66 international female students contacted the research assistant; among them, three were not eligible after screening and one refused to have a blood test for hormones. Finally, 62 were randomly assigned to either an intervention group or a control group, and all of them had finished the follow-ups (Figure 1). The participants were enrolled from 30 countries, with a mean age of 27.87 ± 5.58 years. The majority were single (79%), studying in a master's program (58.1%), and beneficiaries of the Chinese government scholarship. The other socio-demographic characteristics are shown in Table 1. There are no statistically significant differences between the two groups.

**Table 1:** General characteristics and socio-demographics of participants.

**Variables**	**Intervention group (*n* = 31)*N* (%)**	**Control group (*n* = 31)*N* (%)**	***X*^2^ or *Z***	***P*-value**
**Age (years)**
17–26	12 (38.8)	13 (41.9)	0.75^b^	0.76
27–36	15 (48.4)	16 (51.6)		
37–46	4 (12.9)	2 (6.5)		
**Marital status**
Single	23 (74.2)	26 (83.9)	0.87^a^	0.53
Married	8 (25.8)	5 (16.1)		
**Coming from**
Asia	14 (45.2)	15 (48.4)	0.06^a^	1.00
Africa	17 (54.8)	16 (51.6)		
**Major**
Medical Sciences	13 (41.9)	9 (29.0)	2.77^b^	0.63
Engineering	7 (22.6)	9 (29.0)		
Management	7 (22.6)	5 (16.1)		
Economics	1 (3.2)	3 (9.7)		
Language	3 (9.7)	5 (16.1)		
**Program**
Bachelor	13 (41.9)	10 (32.3)	0.92^b^	0.69
Master	17 (54.8)	19 (61.3)		
PhD	1 (3.2)	2 (6.5)		
**Enrollment year**
2019	16 (51.6)	11 (35.5)	1.67^b^	0.45
2018	12 (38.7)	16 (51.6)		
2017	3 (9.7)	4 (12.9)		
**Tuition covered by**
Chinese government	25 (80.6)	26 (83.9)	0.11^a^	1.00
Themselves	6 (19.4)	5 (16.1)		
**Official language**
English	10 (32.3)	8 (25.8)	0.31^a^	0.78
Others	21 (67.7)	23 (74.2)		
**Been to China before studying here**
Yes	3 (9.7)	2 (6.5)	0.21^a^	1.00
No	28 (90.3)	29 (93.5)		
**Having any health insurance in China**
Yes	11 (35.5)	16 (51.6)	1.64^a^	0.30
No	20 (64.5)	15 (48.4)		

a Chi-square test was used to compare data between intervention and control groups.

b Fisher's exact test was used to compare data between intervention and control groups.

### 3.2 Comparison of the menstrual symptoms at the baseline

The scores of MSQ at the baseline between the intervention group (63.93 ± 13.27)and the control group (63.48 ± 16.93)were comparable. In addition, for the three subscales of MSQ, the scores were comparable, with premenstrual symptoms (intervention group: 26.81 ± 7.56 vs. control group: 27.42 ± 7.57), psychophysiological discomfort (17.52 ± 3.60 vs. 16.52 ± 5.22), and menstrual pain (19.61 ± 5.32 vs. 19.55 ± 5.16). There are no statistically significant differences among the total scores of the MSQ scale, the three subscales of MSQ, and the individual items of the MSQ scale between the intervention group and the control group, which are presented in Table 2 and Supplementary Table S1.

**Table 2:** Comparison of the changes in scores of menstrual symptoms between the two groups.

**Items**	**Intervention group mean** ±**SD**			**Control group mean** ±**SD**			**F** ^**¶**^ ***P*** ^**¶**^ **(between groups among the three time points)**			***t*^§^*P*^§^(between group difference at T0)**
	**T0**	**T1**	**T2**	**T0**	**T1**	**T2**	**Groups** ^a^	**Times** ^b^	**Groups BY Times** ^c^	
MSQ (total score)	63.93 (13.27)	53.93 (10.47)	47.83 (8.71)	63.48 (16.93)	64.54 (16.30)	65.12 (16.86)	19.49 (0.00)^**^	4.13 (0.01)^*^	6.23 (0.00)^**^	0.11 (0.90)
**Subscale of MSQ**										
1. Premenstrual symptoms	26.81 (7.56)	21.00 (5.01)	17.07 (3.33)	27.42 (7.57)	28.13 (7.51)	28.42 (7.56)	44.70 (0.00)^**^	7.09 (0.00)^**^	10.77 (0.00)^**^	−0.34 (0.73)
2. Psychophysiological discomfort	17.52 (3.60)	15.45 (3.29)	14.74 (3.29)	16.52 (5.22)	16.84 (5.20)	17.13 (5.22)	2.06 (0.15)	1.05 (0.35)	2.43 (0.09)	0.88 (0.38)
3. Menstrual pain	19.61 (5.32)	17.48 (4.72)	16.03 (4.53)	19.55 (5.16)	19.58 (4.92)	19.58 (5.14)	6.50 (0.01)^*^	1.99 (0.14)	2.07 (0.13)	0.05 (0.96)

SD, standard deviation. ^§^Independent sample t-test among the intervention group and control group for the baseline only.

^¶^Multivariate analysis of variance (MANOVA)between the intervention group and control group among the three time points.

^*^P < 0.05.

^**^P < 0.01.

a Degree of freedom for groups was 1.

b Degree of freedom for times was 2.

b Degree of freedom for groups by times was 2.

### 3.3 Menstrual symptom changes during the study period

**Figure 2:**
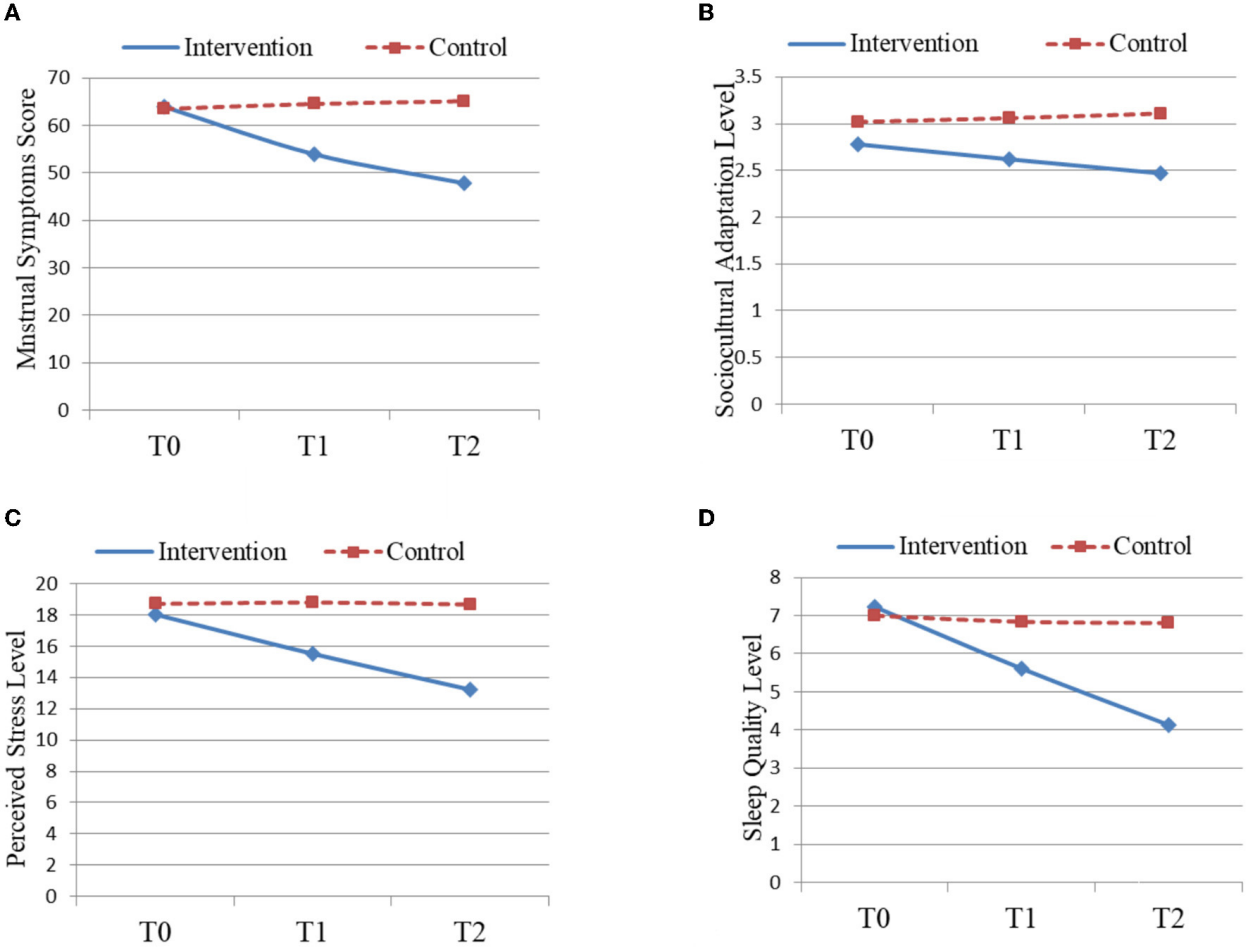
Comparison of the changes among the study variables between the intervention and control groups during the study period **(A–D)**.

The results of the MANOVA test showed traditional Chinese Baduanjin exercise reduced the total score of MSQ among international female students studying in China (intervention group: 47.83 ± 8.71 vs. control group: 65.12 ± 16.86; *F* = 19.49, *P* < 0.01), which are presented in Table 2 and Figure 2. The total score of MSQ for participants in the intervention group decreased with the increase in follow-up time (*F* = 4.13, *P* < 0.05), as presented in Figure 2 and Table 2. In addition, results of the MANOVA indicated statistically significant decreases in scores for the two subscales: premenstrual symptoms (intervention group: 17.07 ± 3.33 vs. control group: 28.42 ± 7.56; *F* = 44.70, *P* < 0.01)and menstrual pain (intervention group: 16.03 ± 4.53 vs. control group: 19.58 ± 5.14; *F* = 6.50, *P* < 0.05; Table 2). However, no statistically significant difference was found in the subscale of psychophysiological discomfort between the intervention (14.74 ± 3.29)and control groups (17.13 ± 5.22; *F* = 2.06, *P* > 0.05; Table 2). The differences in the 24 individual items of the MSQ between the intervention and control groups were compared, and the results are presented in Supplementary Table S1.

### 3.4 Comparison of the secondary outcomes

There were no statistically significant differences in the baseline scores among scores of sociocultural adaptation (SCAS), stress level (PSS), sleep quality (PSQI), blood progesterone hormone, and blood estrogen hormone between the two groups (*P* > 0.05). The results of the MANOVA test showed traditional Chinese Baduanjin exercise reduced the scores of the sociocultural adaptation level (intervention group: 2.47 ± 0.71 vs. control group: 3.11 ± 0.60; *F* = 21.67, *P* < 0.01), perceived stress level (intervention group: 13.23 ± 5.73 vs. control group: 18.67 ± 4.56; *F* = 15.95, *P* < 0.01), and sleep quality (intervention group: 4.13 ± 1.94 vs. control group: 6.806 ± 2.16; *F* = 12.90, *P* < 0.01)among international female students studying in China (Table 3). In addition, compared to participants in the control group, the intervention significantly improved the blood progesterone hormone (intervention group: 170.33 ± 87.57 vs. control group: 135.10 ± 85.32; *F* = −5.21, *P* < 0.01)and blood estrogen hormone (intervention group: 15.30 ± 10.86 vs. control group: 8.30 ± 10.35; *F* = −7.35, *P* < 0.01)for participants in the intervention group (Table 3).

**Table 3:** Comparison of the secondary outcomes during the study period.

**Items**	**Intervention group mean** ±**SD**			**Control group mean** ±**SD**			***F** ^**¶**^ ***P*** ^**¶**^* **(between groups among the three time points)**			***t* ^§^*P* ^§^(between groups difference at T0)**
	**T0**	**T1**	**T2**	**T0**	**T1**	**T2**	**Groups** ^a^	**Times** ^b^	**Groups BY Times** ^c^	
SCAS	2.78 (0.73)	2.62 (0.69)	2.47 (0.71)	3.02 (0.54)	3.06 (0.54)	3.11 (0.60)	21.67 (0.00)^**^	0.41 (0.66)	1.55 (0.21)	−1.42 (0.15)
PSS	18.03 (6.25)	15.52 (5.99)	13.23 (5.73)	18.71 (4.79)	18.80 (4.54)	18.67 (4.56)	15.95 (0.00)^**^	3.16 (0.04)^*^	3.08 (0.04)^*^	−0.47 (0.63)
PSQI	7.23 (2.81)	5.61 (2.26)	4.13 (1.94)	7.000 (2.35)	6.839 (2.33)	6.806 (2.16)	12.90 (0.00)^**^	7.76 (0.00)^**^	6.03 (0.00)^**^	0.34 (0.73)
							***t** ^Δ^ **P*** **(between Intervention group)**	***t** ^Δ^ **P*** **(between Control group)**	***t*** ^§^***P*** ^§^**(between groups difference at T2)**	***t*** ^§^***P*** ^§^**(between groups difference at T0)**
Blood progesterone hormone	9.37 (11.61)	–	15.30 (10.86)	7.98 (10.78)	–	8.30 (10.35)	−7.35 (0.00)^**^	−1.76 (0.08)	2.59 (0.01)^*^	0.48 (0.62)
Blood estrogen hormone	138.64 (82.08)	–	170.33 (87.57)	134.18 (85.14)	–	135.10 (85.32)	−5.21 (0.00)^**^	−1.64 (0.11)	1.60 (0.11)	0.21 (0.83)

SD, standard deviation. ^**§**^Independent sample t-test.

^**Δ**^Paired sample t-test.

^¶^Multivariate analysis of variance (MANOVA)between the intervention group and control group among the three time points.

^*^P < 0.05.

^**^P < 0.01.

a Degree of freedom for groups was 1.

b Degree of freedom for times was 2.

c Degree of freedom for groups by times was 2.

### 3.5 Intervention adherence

As for the intervention maintenance, two-thirds of participants completed all planned practice sessions for the two phases of intervention, while the remaining one-third completed more than 85% of planned practice sessions. None of them reported an adverse event.

## 4 Discussion

This randomized controlled trial indicated that the traditional Chinese Baduanjin exercise is acceptable and could reduce not only the menstrual symptoms but also the acculturation-related stress and sleep problems for international female students. The gestures and movements of the Baduanjin exercise are simple and easy to learn; thus, the adherence to the Baduanjin exercise is optimal, with all of them having completed at least 85% of the practice sessions without any adverse events, which indicates the participants' satisfaction with the practice and the ease and safety of the exercise.

Our results showed that Baduanjin exercise has a positive effect on reducing menstrual symptoms over time, and the longer the practice, the fewer the menstrual symptoms. Many symptoms have been relieved after practicing Baduanjin for 3–6 months, such as fatigue, constipation, weight gain, abdominal discomfort and bloating, backaches, and irritability. Previous studies demonstrated that physical symptoms were often associated with fluctuations in the estrogen, progesterone, and serotonin levels among females with menstrual symptoms (17), and our study results also confirmed such an association and showed that the progesterone and estrogen levels also increased along with the decrease in menstrual symptom levels. The possible mechanism might be that Baduanjin exercises lower renin-angiotensin activity, increase progesterone and estrogen, and decrease serum aldosterone levels, resulting in symptomatic improvement (18).

Regarding the subscales, our study showed that Baduanjin could significantly reduce premenstrual symptoms and menstrual pain, which were consistent with previous studies (43, 44). Our study did not show a significant effect on the subscale of psychophysiological discomfort. However, we can see a decreasing trend in the total score of this subscale in the intervention group compared with the control group, and it might take longer intervention time to detect the statistical difference between the two groups. Similarly, Zhang's study also showed that Baduanjin exercise was more effective in reducing physical symptoms than psychological symptoms (30).

Several studies have found that university students reported higher levels of perceived stress due to regular examinations, specified deadlines, and the competition to perform well (45). Our results showed that university students' stress levels and psychological wellbeing could be improved through Baduanjin exercise. As Baduanjin exercise allows participants to practice body, breath, and mind control (46)through slow, continuous movements and steady postures, which highly demand mental focus and breathing control (47). Another rationale might lie in the distraction hypothesis (48)because the Baduanjin exercise is intended to induce practitioners to focus on the moment and forget about moments of stressful events. In addition, the positive impact on reducing stress by practicing Baduanjin can result in the individual's participation in a new interesting activity that develops the self and enhances social interactions, and this is consistent with the social interaction hypothesis that assumes the occurrence of mutual support between the individuals participating in the exercise (49). Moreover, the biological theory states that physical activity increases monoamines synaptic transmission, including serotonin, adrenaline, and dopamine, which are hypothesized to function as anti-depressives (50).

Sleep is a biological regulatory process essential to the nervous system and basic human needs, including metabolism, immunity, and hormonal balance (51, 52). Sleep disturbance impacts a considerable number of people (53), in particular, university students and international students who may experience sleep problems due to academic pressures, environmental changes, and housing conditions, which leads to a deterioration in health and academic performance (54, 55). The American Academy of Sleep Medicine recommends regular exercise to maintain healthy sleep (56). Systematic reviews have reported a positive effect of exercise on sleep duration and quality (57, 58), which our study has also confirmed. The beneficial effect of the Baduanjin exercise on sleep quality may be related to several properties of Baduanjin: modulating the mind and spirit, enhancing respiratory function via promoting a calm state and relaxation, and encouraging regular and deep breathing (59). In addition, practicing Baduanjin exercise could stimulate metabolism, reinforce Qi (energy)and blood circulation, mitigate stress, and enhance spiritual life (60). Baduanjin could enhance sleep quality by increasing melatonin, an influential hormone regulating sleep-wake rhythm and the autonomic nervous system (61).

### 4.1 Study limitations

Some potential limitations are inevitable in this experimental study. First, this study was conducted at only two universities in one province, so the results might not be representative of all international female students in China. Second, the symptom assessment was self-reported, and recall bias and social desirable bias might exist. Third, due to financial limitations, we did not have a wider laboratory evaluation of hormones, such as luteinizing hormone (LH)and follicle stimulating hormone (FSH)during all the study phases. Fourth, contamination might exist when participants in the EG group and the CG group knew each other. Participants in the CG group could learn Baduanjin from those in the EG group. Fifth, the actual intensity of the Baduanjin exercise was not evaluated in this study. Finally, confounding factors, such as smoking status, body mass index, alcohol consumption, the intensity level of other daily activities, and exposure to stressful events, were not controlled during the program period.

### 4.2 Study implications

Despite the limitations, our results have practical implications. The results of the current study improve our knowledge regarding the safety, acceptability, and beneficial effects of Baduanjin practice on menstrual physical and psychological symptoms for international female students who endure menstrual symptoms due to acculturation stress. This practice provides an additional option for non-pharmacological treatment and self-care to maintain menstrual health and psychological health for international students during the acculturation period.

## 5 Conclusion

Our findings indicate that regular Baduanjin exercise could be an effective, safe, and acceptable form of exercise for reducing menstrual symptoms and enhancing the wellbeing of female students studying in China. Qualitative studies are needed in the next step to better understand their feelings and perceptions, barriers, and facilitators about practicing such traditional Chinese exercises for international students.

## Data availability statement

The original contributions presented in the study are included in the article/Supplementary material, further inquiries can be directed to the corresponding authors.

## Ethics statement

The studies involving humans were approved by Institutional Review Board of behavioral and Nursing Research in Xiangya School of Nursing of Central South University (Approval No. E2020193). The studies were conducted in accordance with the local legislation and institutional requirements. The participants provided their written informed consent to participate in this study.

## Author contributions

AA: Conceptualization, Formal analysis, Investigation, Methodology, Validation, Writing—original draft, Writing—review & editing. HA: Investigation, Methodology, Validation, Writing—review & editing. CZ: Data curation, Formal analysis, Methodology, Writing—review & editing. WP: Investigation, Methodology, Validation, Writing—review & editing. XL: Conceptualization, Formal analysis, Funding acquisition, Investigation, Methodology, Project administration, Supervision, Writing—review & editing.
