# Supplementary material for: Impact of traditional Chinese Baduanjin exercise on menstrual health among international female students studying in China: a randomized controlled trial
Source: Front Public Health. 2024 Feb 7;12:1259634. doi: 10.3389/fpubh.2024.1259634 (PMC10879288; doi:10.3389/fpubh.2024.1259634)
Supplement: Supplementary file 1 [file Table_1.DOC]

Supplementary table 1. Comparison of the changes in scores of menstrual symptoms item by item between the two groups

| **Items** | **Intervention Group**  **Mean ± SD** | | | **Control Group**  **Mean ± SD** | | | ***F*^⁋^*P*^⁋^ (Between groups among the three time points)** | | | ***t*^§^ *P*^§^ (Between Group difference at T0)** |
| --- | --- | --- | --- | --- | --- | --- | --- | --- | --- | --- |
|  | T0 | T1 | T2 | T0 | T1 | T2 | **Groups** | **Times** | **Groups BY Times** |  |
| **Subscale: Premenstrual symptoms** | | | | | | | | | | |
| 1.Feel irritable a few days before the period | 3.06 (1.36) | 2.26 (0.99) | 1.61 (0.84) | 3.19 (1.04) | 3.16 (1.29) | 3.32 (1.32) | 28.78 (0.00) ****** | 5.14 (0.00) ****** | 7.17 (0.00) ****** | - 0.41 (0.67) |
| 3.Feel depressed for several days before the period | 2.84 (1.26) | 1.87 (0.92) | 1.58 (0.76) | 2.87 (1.11) | 2.94 (1.15) | 2.84 (1.00) | 25.94 (0.00) ****** | 6.15 (0.00) ****** | 6.10 (0.00) ****** | - 0.10 (0.91) |
| 4.Had abdominal pain which begins one day before the period | 2.81 (1.22) | 2.52 (1.20) | 2.23 (1.14) | 3.32 (1.04) | 3.39 (1.08) | 3.42 (1.14) | 26.28 (0.00) ****** | 0.69 (0.50) | 1.36 (0.26) | - 1.78 (0.07) |
| 5.Feel fatigued for several days before the period | 3.00 (1.06) | 2.84 (1.09) | 2.48 (1.12) | 3.35 (1.08) | 3.42 (1.02) | 3.29 (1.07) | 13.50 (0.00) ****** | 1.29 (0.27) | 0.68 (0.50) | - 1.30 (0.19) |
| 11.Have backaches for several days before the period | 2.97 (1.19) | 1.90 (0.87) | 1.45 (0.62) | 2.68 (1.19) | 2.65 (1.30) | 2.77 (1.35) | 12.92 (0.00) ****** | 6.81 (0.00) ****** | 8.22 (0.00) ****** | 0.95 (0.34) |
| 13.Feel that the breasts tenderness a few days before the period | 2.81 (1.25) | 2.68 (1.04) | 2.48 (1.15) | 2.26 (1.03) | 2.23 (1.08) | 2.16 (1.09) | 7.30 (0.00) ****** | 0.56 (0.57) | 0.16 (0.85) | 1.88 (0.06) |
| 16.Gain weight before the period | 1.97 (1.27) | 1.23 (0.49) | 1.00 (0.00) | 1.94 (1.23) | 2.16 (1.21) | 2.29 (1.32) | 22.50 (0.00) ****** | 1.52 (0.22) | 6.58 (0.00) ****** | 0.10 (0.91) |
| 20.Have abdominal discomfort more than one day before the period | 2.58 (1.08) | 1.97 (0.83) | 1.42 (0.62) | 2.90 (1.19) | 3.10 (1.32) | 3.03 (1.22) | 41.90 (0.00) ****** | 3.61 (0.02) ***** | 5.69 (0.00) ****** | - 1.11 (0.27) |
| 22.Feel your abdominal bloated before the period | 2.68 (1.13) | 2.00 (0.85) | 1.35 (0.55) | 2.29 (1.27) | 2.61 (1.14) | 2.65 (1.19) | 10.65 (0.00) ****** | 3.33 (0.03) ***** | 9.90 (0.00) ****** | 1.26 (0.21) |
| 24.Have headaches for a few days before the period | 2.10 (0.90) | 1.74 (0.72) | 1.45 (0.72) | 2.61 (1.28) | 2.48 (1.33) | 2.65 (1.35) | 26.06 (0.00) ****** | 1.36 (0.26) | 1.55 (0.21) | - 1.82 (0.07) |
| **Subscale: Psychophysiological discomfort** | | | | | | | | | | |
| 7.Take a prescription medication for the pain during the period | 2.06 (1.20) | 2.00 (1.26) | 2.00 (1.23) | 2.13 (1.17) | 2.03 (1.11) | 2.06 (1.18) | 0.09 (0.76) | 0.08 (0.92) | 0.00 (0.99) | - 0.21 (0.83) |
| 8.Feel weak during the period | 3.26 (0.99) | 2.35 (0.98) | 1.90 (1.04) | 3.16 (0.93) | 3.19 (1.01) | 3.10 (1.01) | 19.41 (0.00) ****** | 7.96 (0.00) ****** | 6.91 (0.00) ****** | 0.39 (0.69) |
| 9.Feel tense and nervous before the period | 2.94 (1.06) | 2.39 (1.14) | 2.39 (1.17) | 2.55 (1.15) | 2.65 (1.14) | 2.45 (1.15) | 0.02 (0.89) | 1.31 (0.27) | 1.31 (0.27) | 1.37 (0.17) |
| 10.Have diarrhea during the period | 2.58 (1.31) | 2.55 (1.33) | 2.58 (1.31) | 2.29 (1.16) | 2.42 (1.23) | 2.52 (1.28) | 0.74 (0.38) | 0.12 (0.88) | 0.13 (0.87) | 0.92 (0.35) |
| 15.Heat make you comfortable, such as use a hot water bottle during the period | 3.29 (1.13) | 3.29 (1.13) | 3.23 (1.05) | 2.71 (1.29) | 2.68 (1.30) | 2.81 (1.37) | 9.02 (0.00) ****** | 0.01 (0.98) | 0.11 (0.89) | 1.87 (0.06) |
| 17.Had constipation during the period | 1.65 (0.70) | 1.19 (0.40) | 1.00 (0.00) | 1.71 (1.00) | 1.65 (1.01) | 1.84 (1.24) | 13.38 (0.00) ****** | 1.94 (0.14) | 3.28 (0.04) ***** | - 0.29 (0.77) |
| 23.Feel nauseous during the period | 1.74 (0.81) | 1.68 (0.79) | 1.65 (0.79) | 1.97 (1.19) | 2.23 (1.28) | 2.35 (1.51) | 9.34 (0.00) ****** | 0.28 (0.75) | 0.77 (0.46) | - 0.86 (0.38) |
| **Subscale: Menstrual pain** | | | | | | | | | | |
| 2.Have cramps during the period | 3.32 (1.22) | 2.90 (1.04) | 2.42 (1.05) | 3.58 (1.28) | 3.61 (1.25) | 3.52 (1.33) | 15.15 (0.00) ****** | 2.53 (0.08) | 1.88 (0.15) | - 0.81 (0.42) |
| 6.Know that the period coming by looking at the calendar | 2.71 (1.34) | 2.65 (1.33) | 2.58 (1.33) | 3.26 (1.15) | 3.29 (1.16) | 3.23 (1.14) | 11.20 (0.00) ****** | 0.07 (0.93) | 0.03 (0.96) | - 1.72 (0.09) |
| 12.Take Aspirin for the pain during the period | 2.16 (1.21) | 1.35 (0.60) | 1.06 (0.25) | 2.10 (1.10) | 2.06 (1.09) | 2.23 (1.11) | 18.16 (0.00) ****** | 4.61 (0.01) ***** | 6.41 (0.00) ****** | 0.21 (0.82) |
| 14.Feel any pain in the lower back, abdomen during the period | 3.26 (1.18) | 3.19 (1.19) | 3.13 (1.25) | 2.87 (1.02) | 2.84 (1.09) | 2.81 (0.94) | 4.65 (0.03) ***** | 0.12 (0.89) | 0.01 (0.98) | 1.37 (0.17) |
| 18.Have pain spasms during the period | 2.55 (1.23) | 2.32 (1.13) | 2.10 (1.19) | 2.61 (1.02) | 2.58 (1.02) | 2.65 (1.14) | 3.08 (0.08) | 0.54 (0.58) | 0.72 (0.48) | - 0.22 (0.82) |
| 19.Have dull continuous pain during the period | 2.55 (1.06) | 2.55 (1.06) | 2.58 (1.08) | 2.52 (0.99) | 2.61 (1.11) | 2.52 (1.02) | 0.00 (0.94) | 0.03 (0.96) | 0.06 (0.94) | 0.12 (0.90) |
| 21.Have backaches during the period | 3.06 (1.06) | 2.52 (1.15) | 2.16 (1.29) | 2.61 (1.14) | 2.58 (1.17) | 2.65 (1.22) | 0.03 (0.85) | 2.20 (0.11) | 2.45 (0.08) | 1.60 (0.11) |

Sd: standard deviation; **^§^**: Independent Sample t test among the intervention group and control group for the baseline only; **^⁋^:** Multivariate analysis of variance (MANOVA) between the intervention group and control group among the three time points; ***:** represent p < 0.05**; **:** represent p < 0.01**.**
